# “You don’t want to lose that trust that you’ve built with this patient…”: (Dis)trust, medical tourism, and the Canadian family physician-patient relationship

**DOI:** 10.1186/s12875-015-0245-6

**Published:** 2015-02-25

**Authors:** Valorie A Crooks, Neville Li, Jeremy Snyder, Shafik Dharamsi, Shelly Benjaminy, Karen J Jacob, Judy Illes

**Affiliations:** Department of Geography, Simon Fraser University, 8888 University Drive, Burnaby, BC V5A 4X9 Canada; Faculty of Health Sciences, Simon Fraser University, 8888 University Drive, Burnaby, BC V5A 4X9 Canada; Faculty of Medicine, University of British Columbia, Vancouver, BC Canada

**Keywords:** Trust, Physician-patient relationship, Medical tourism, Canada

## Abstract

**Background:**

Recent trends document growth in medical tourism, the private pursuit of medical interventions abroad. Medical tourism introduces challenges to decision-making that impact and are impacted by the physician-patient trust relationship—a relationship on which the foundation of beneficent health care lies. The objective of the study is to examine the views of Canadian family physicians about the roles that trust plays in decision-making about medical tourism, and the impact of medical tourism on the therapeutic relationship.

**Methods:**

We conducted six focus groups with 22 family physicians in the Canadian province of British Columbia. Data were analyzed thematically using deductive and inductive codes that captured key concepts across the narratives of participants.

**Results:**

Family physicians indicated that they trust their patients to act as the lead decision-makers about medical tourism, but are conflicted when the information they are managing contradicts the best interests of the patients. They reported that patients distrust local health care systems when they experience insufficiencies in access to care and that this can prompt patients to consider going abroad for care. Trust fractures in the physician-patient relationship can arise from shame, fear and secrecy about medical tourism.

**Conclusions:**

Family physicians face diverse tensions about medical tourism as they must balance their roles in: (1) providing information about medical tourism within a context of information deficits; (2) supporting decision-making while distancing themselves from patients’ decisions to engage in medical tourism; and (3) acting both as agents of the patient and of the domestic health care system. These tensions highlight the ongoing need for reliable third-party informational resources about medical tourism and the development of responsive policy.

## Background

People have long traveled in search of improved health and wellbeing, whether going to the neighborhood doctor’s office for a routine appointment or going abroad to visit places known for their healing properties. In recent years, the popularity of a particular global health care mobility known as medical tourism has risen significantly. Medical tourism involves patients’ independent pursuit of health care abroad, and is distinguished from arranged cross-border care as it is privately paid for [1-3]. The industry is global in scope, with revenues reported to be growing rapidly [4-6]. Hospitals, clinics, and service providers from an ever-expanding list of countries seek to attract international patients^a^, and market directly to them through the Internet and via intermediaries such as facilitators [7,8]. Patients can purchase a range of procedures from medical tourism facilities, including unproven interventions that are unavailable or illegal in their home countries, such as stem cell interventions, and routine procedures that are readily available where they live, such as orthopaedic surgeries [9,10].

Promotional materials aimed at international patients, such as websites and brochures, cast medical tourism as a highly positive and trustworthy practice [11,12]. Much scholarly research points to the lack of balanced information available to prospective medical tourists, which in turn fuels unfettered expectations at the cost of informed hopes [12,13]. Media-generated hype further complicates the landscape of endorsement, portraying some interventions as imminent and risk-free cures, and often focusing on the financial costs of procedures rather than possible risks [14-16]. Meanwhile, this often hype-driven, biased information is commonly used by prospective medical tourists when deciding on whether or not to pay for private care abroad [17]; though other sources of information, such as the opinions of their regular physicians at home, are also taken into consideration [18,19].

The medical tourism industry operates vigorously despite the veil of concern and criticism that surrounds it. Some of these concerns pertain to the impact that the industry has on destination countries, in which the highly privatized industry draws resources and health workers away from public health systems [1,20]. Other concerns focus on the impact that the industry has on the home countries of medical tourists, such as the public health risks associated with infections acquired abroad that may spread locally [21,22], the burden of providing either simple or complex follow-up care [23-25], and the impact that patients’ decisions to go abroad for care have on the ongoing relationships they hold with their regular physicians [19,26]. Despite these and other concerns, promotional materials aimed at international patients cast medical tourism as a highly positive and trustworthy practice, which has led some researchers and health professionals to speculate that prospective medical tourists are not being provided with the information they need to make truly informed decisions [12,27,28].

Recent studies provide examples of family physicians declining to care for returning medical tourists or refusing to coordinate their follow-up care, citing both the weight on the personal-professional relationship, as well as the difficulty of integrating any acquired medical benefits and harms into the responsibilities they hold towards these patients [18,29]. Physician-patient relationships are formed over time on the “expectation that the other person will behave in a way that is beneficial, or at least not harmful, and allows for risks to be taken based on this expectation” (p.148) [30]; in other words, they are formed on a basis of trust in the fiduciary relationship. Physicians’ trust in patients in turn enhances patients’ trust in their physicians, and such mutual trust enhances cooperation [31,32]. A recent systematic review found that trusting relationships facilitate shared decision-making, open communication, minimization of patients’ fears, and better adherence to health advice [33]. The formation of a trusting relationship between physicians and patients is thus the foundation upon which truly beneficent health care can be built, and thus it raises cause for concern if a patient’s decision to participate in medical tourism threatens its development or continuance.

In this article we examine the roles of trust and distrust in decision-making, information exchanges, and health care in the context of Canadian family physicians who are faced with addressing questions about medical tourism from their patients, or who are treating former medical tourists in their practices. We conceive of trust as a belief in the soundness of a person, issue, or information source while distrust is the lack of such belief. In some cases these patients look to their regular family physicians to help them in the decision-making process, to prepare their medical records to be taken abroad, to prescribe medications that destination facilities want patients to have on-hand, to review their medical records from abroad, and to refer them for local follow-up care. In other cases, patients opt not to tell their family physicians about their participation in medical tourism until returning home, if at all, out of concern that these physicians will be unsupportive or judgmental [18,19,34]. Building on past research in this area, we advance the understanding of why and how the established physician-patient relationship, a relationship founded on trust, is impacted by a patient’s decision to participate in medical tourism.

## Methods

In 2011, we held six focus groups in six different cities with family physicians in the Canadian province of British Columbia to explore what they identified as the implications of the global health service practice of medical tourism on their practice. The cities selected for data collection for this qualitative exploratory study varied in size and spanned all five of British Columbia’s regional health authorities responsible for health service administration and delivery. British Columbia was selected as the province of data collection because existing research and media coverage have shown that many medical tourism facilitation companies operate there and that some residents are opting to engage in medical tourism [8,35]. Family physicians practicing in this province, therefore, are likely to encounter former medical tourists and questions about medical tourism in their practices in addition to being involved in discussions about medical tourism with colleagues.

### Participant recruitment

Following approval from the Research Ethics Board at Simon Fraser University, we obtained a list of all family physicians practicing in the cities selected to host focus groups from the British Columbia College of Family Physicians directory. We faxed a letter of invitation to each family medicine practice or family physician listed in the directory in each city, along with details about the study and how to express interest in participating. The letter explained that having seen significant numbers of medical tourists in their practices was not a pre-requisite for participating in the study. The letter also provided a brief description of the team’s previous research on the local and global equity and ethical impacts of medical tourism and a link to the research website. We also requested that recipients share the letter with colleagues to increase the number of potential participants receiving study information.

Family physicians interested in participating in a focus group were asked to call a toll-free number or send an e-mail to request more information. After such expressions of interest were received a team member followed up to ensure eligibility (i.e., that the person was indeed practicing family medicine in one of the 6 cities of focus) and to relay information about the focus group time and location. Reminder e-mails or phone calls, depending on the participant’s preference, were sent to those who had agree to participate a few days in advance of the focus group and again on the day of the meeting.

### Data collection

Six physician focus groups lasting from 1.5 to 2 hours each were hosted in a meeting room at a centrally located hotel in each city. The participants each signed a written consent form at the start of the meeting. Two co-moderators and a note-taker ran the groups, who were drawn from VAC, JS, SD, and two research assistants working with VAC and JS. The lead co-moderator facilitated the group while the second co-moderator (re)focused the discussion when necessary. One co-moderator was always a faculty member with previous qualitative data collection experience (VAC [a health services researcher], JS [a bioethicist] and SD [a global health researcher]) while the other was a highly qualified graduate student who had worked with the team on previous studies. All investigators had previously studied medical tourism, including the graduate students, and had publication records in the field.

Probes designed to explore participants’ experiences with and perspectives about medical tourism guided the focus group discussions. The probes were developed based on an extensive review of the international medical tourism literature [25,35], as well as insights gleaned from a previous study that identified family physicians as sometimes being involved in Canadian patients’ decision-making around medical tourism [17]. Given the exploratory nature of this study, the probes were intentionally broad and inquired into: existing knowledge of medical tourism and experiences with medical tourists in their practices (e.g., what is medical tourism? Tell us what you know about it based on your experience); perceived and experienced impacts of medical tourism on the physician-patient relationship (e.g., asking participants to talk about information sharing and exchange, assisting with decision-making, patient education, and advising for or against medical tourism); provision of follow-up care for returning medical tourists (e.g., asking participants to talk about continuity of care, care quality, patient risks, and access to follow-up care); and the impacts of medical tourism for local health care more broadly (e.g., asking participants to talk about why patients are going abroad as medical tourists). Following conventional methods for focus groups, the probes were intended to stimulate discussion while participants drove the scope and breadth of the conversations.

### Data analysis

Verbatim transcripts of the focus groups were produced from digital recordings. Following completion of data collection, the lead investigators independently reviewed all transcripts and a team meeting was held to identify emerging themes for further analysis. A coding scheme was created using deductive and inductive codes to capture key concepts. The transcripts were then uploaded into the qualitative data management program NVivo and coded using the consensus-based scheme. To enhance consistency, a single investigator was the primary coder while another investigator provided confirmation on interpretation wherever necessary.

Three thematic findings for full analysis were identified through the process of independent transcript review and team discussion. Coded data pertaining to each thematic analysis were extracted from the main dataset and reviewed in detail for key emerging findings. The foci of these three analyses were: (1) the roles and responsibilities of family physicians towards patients engaging in medical tourism, within which we identified participants’ understandings of pre- and post-trip roles and responsibilities towards medical tourists in their practices; (2) the challenges medical tourism poses to family physician involvement in informed decision-making, wherein we examined issues such as the shifting of responsibility for health outcomes from the family physician to the patient in light of the decision to go abroad and the ethical tensions that family physicians face in treating medical tourists in their practices; and, (3) family physicians’ perspectives about the complex roles that trust plays in their interactions with prospective or former medical tourists. This article examines the third, trust-focused analysis. The other two have been published elsewhere [18,19].

All authors reviewed coding extracts related to the trust-focused analysis independently and as a group to achieve consensus on analytic scope. The final analysis was organized around the sub-themes of trust, distrust, and implications for trust in the physician-patient relationship. Wherever possible direct quotes from participants are used to illustrate findings. The quotes were selected from the data extracts reviewed by all authors.

## Results

A total of 22 family physicians participated across the 6 focus groups. Participants had been engaged in practice for an average of 23 years. Of the 22 participants, 20 had direct experience caring for medical tourists in their practices while all had heard about medical tourism prior to participating in the focus group. The number of former medical tourists they had encountered varied from 1 to approximately 90, with a median of 6. In the remainder of this section we examine the ways in which participants raised issues of trust and distrust in the focus group discussions, and ultimately their reflections on how patients’ decisions to engage in medical tourism may impact the ongoing trusting physician-patient relationship.

### Issues of trust

Although the concept of trust was not a specific probe, issues relevant to trust emerged as a topic of concern in each of the focus groups. There was consensus among the participants that it is important for family physicians to trust their patients’ abilities to make beneficent health-related decisions about medical tourism and to be the lead decision-makers around treatment abroad. As one participant pointed out, “*they [the patients] have to make the decision*.” Participants commonly did not believe that family physicians should be central to patients’ decision-making processes given all the uncertainties surrounding medical tourism, and instead family physicians should “*give them [the patients] the information so that they could make an informed decision …”* As one participant stated: *“ I make sure that when they leave [my office] they understand that what I’ve tried to do is help them make an informed decision*.” Participants shared that trust is fostered by answering patients’ questions as best as possible, providing information about which they feel confident, and respecting patient autonomy. However, as we discuss later in greater detail, the formation of such trust is challenged when physicians do not support the choice for medical tourism or are concerned about the safety and efficacy of the treatment being sought.

Participants readily acknowledged that the degree of trust a patient has in a physician or a clinic abroad heavily influences decision-making. The medical tourism industry attempts to foster such trust by trying to ensure that the patient is “*sold by the beauty of it…the recovering on the beach…[and] excellent care*.” Participants further acknowledged that repeated positive experiences with a country’s health system could enhance the sense and development of trust. For example, if patients “*came from India…[or] frequently make trips to…India and they trust the system there*” or are “*from Taiwan, you know the place, you went back there and you get a surgery there*.”

Participants expressed that a patient’s trust in physicians, clinics, or treatments abroad can override common sense. For example, one participant spoke of a patient who obtained repeated treatments for a chronic illness in a private clinic in the United States over a period of several years, but showed no clinical improvement. Nevertheless, the patient continued to undergo treatment because “*she really believes in them [physicians abroad]*.”

### Issues of distrust

There was a sense among the participants across all of the focus groups that the perceived limitations in British Columbia’s health care system led to distrust among some patients and served as an impetus for medical tourism. Patients who do not find the system to be reliable search for care options elsewhere. Such distrust can stem from a multitude of factors, including “*insufficiency in our system*”, “*outdated procedures*”, “*discrepancies [in access to services] across the [provinces]*”, and being “*frustrated by this…feeling that they’re not cared for, they’re not important enough, their conditions aren’t important enough*” because of waiting lists. In fact, participants were concerned that returning medical tourists may foster such distrust in the local health system among others in their networks by talking about “*how much better it can be [abroad] and how much cleaner and more modern the hospitals can be, how the equipment can just be so much more up-to-date*” abroad. Generally, participants felt that the development of such distrust in the system as a whole is understandable, but they were also concerned that it may also make patients vulnerable to exploitation by physicians and clinics abroad that prey on desperation.

While participants made it very clear that they did not want to play a significant role in patients’ decision-making around medical tourism, they recognized that their own degree of (dis)trust in the procedure, clinic, or physician abroad was important to share with patients. They viewed doing so as an opportunity to provide potentially new or unconsidered information, while ideally trusting the patient to ultimately come to a sound decision. As a participant explained, “*I usually look for red flags in the material that they bring in, and if I see that I say ‘look, I’ve got to warn you about this place’*.” One of the most common distrusts discussed across all focus groups pertained to the quality and reliability of pharmaceuticals available at clinics abroad: “*I have no way of knowing that these [drugs being given to patients] are from a reputable manufacturer*.” Participants reported sometimes doing extensive research on clinics and procedures on their own in order to identify concerns they wished to share with their patients, concerns that also shaped their own level of trust in the procedure or clinic abroad. They also indicated that their previous experiences with medical tourists in their practices impacted this level of trust, specifically indicating that they sometimes drew on past experiences with patients’ poor health outcomes in speaking with prospective medical tourists.

### Trust fractures in the physician-patient relationship

Participants indicated that ‘trust fractures’ could occur in the ongoing relationships they have formed with patients as a result of patients’ decisions to pursue medical tourism, including the act of simply considering such international care options. Fractures could occur in cases where physicians did not act on follow-up care orders administered by care providers abroad for any number of reasons. In such cases patients’ expectations are not met and trust erodes because, as one participant explained, “*he [patient] doesn’t think I [physician] have his best interests at heart*.” Trust fractures can also arise when patients are concerned about being judged negatively for their decisions to go abroad, or when they opt not to disclose their decision prior to departure and consequently threaten continuity of care. As one participant explained: “*they [patients] feel, to some extent…ashamed…like in some way uneasy to reveal this information…because of perceived betrayal of the Canadian system [by leaving the public Medicare system to pay for private care elsewhere]*.” In addition to shame, such fear also leads to secrecy because “*they [patients] may be afraid, and…I think in some cases it would be true, because some doctors would say ‘well if you’re going there don’t be coming back here for me to look after you*’.”

Participants indicated that family physicians ought to undertake active measures to mitigate possibilities of fracturing a trusting relationship because of patients’ involvement in or consideration of medical tourism. Approaching conversations with tact and being mindful of patients’ needs and circumstances was seen as important: “*I’m delicate with most of those patients because if I lose my patient [and] my doctor-patient relationship, then I lose everything with that person. So sometimes I’m a little bit, you know, tip-toe-y*.” Another commonly suggested measure focused on providing information and support to patients while being transparent about personal opinions, acknowledging patients’ autonomy and trusting in their abilities to make sound decisions. One participant described this approach as “*com[ing] alongside*” the patient, while another emphasized that they “*didn’t push them, didn’t pull them, I just helped them to make that decision*.” Other measures undertaken by participants included: communicating with physicians abroad to facilitate information sharing; undertaking additional, but not overly time-consuming, research so as to enhance or augment the patient’s existing information; and avoiding making judgmental statements about medical tourism with patients. Such measures were also thought to aid in avoiding furthering patients’ distrust in domestic health care.

## Discussion

Across the six focus groups held with family physicians in British Columbia, issues of trust and distrust emerged in ways that ultimately complicate the physician-patient relationship. We heard about the importance of respecting patient autonomy in decision-making about medical tourism, concerns about the risks to the patient as well as the therapeutic relationship and noted measures that physicians undertake to protect against trust fractures. The participants with whom we spoke were particularly concerned that patients’ distrust in British Columbia’s health care system may push them to consider participating in medical tourism. Other research also confirms this concern, wherein interviews with former medical tourists from across Canada found that inequities and limitations in the domestic health system were cited by these individuals as a fair justification for their decisions to go abroad for care [36]. Even if Canadians do feel justified in participating in medical tourism because of perceived limitations of the domestic health system, our findings show that this can influence the trust in the physician-patient relationship.

Our findings point to a number of tensions that family physicians must negotiate when dealing with intended or former medical tourists in their practice, and especially in order to not cause a trust fracture in the established relationship. We highlight the three most significant here. First, participants wanted to support patients’ decision-making about medical tourism, but there were limits to and limitations on that support. A key reason for this tension is participants’ distrust in the quality of information available about destination clinics and physicians, particularly when there are few reliable sources of information they can turn to when consulting with patients who are considering medical tourism. In fact, much existing research attention has been given to the lack of third party or neutral information available to those considering medical tourism [6,28,37,38].

Second, while participants expressed a desire to support patients’ decision-making about medical tourism and to trust in their decisions, they also desired to be distanced from the decision itself but not the patient. Participants typically did not want to be seen as endorsing destination physicians or clinics. Instead, they wanted to be open with patients about their own reservations, and they wanted to offer support where appropriate and ultimately trust in their patients’ decision-making abilities; but they did not want to take on significant responsibility in the decision-making process.

Third, significant tension exists between participants’ roles as agents of the patient and their roles as agents of British Columbia’s public health care system. This tension left some participants uncertain about the responsibilities they hold towards medical tourists in their practice, an issue that has been examined elsewhere in some depth [18,19,34]. Arising from this tension is the concern that a lack of shared goals between both parties can compromise trust in the physician-patient relationship, which is an issue that has been raised in the scholarly literature [26]. The findings shared above point to the fact that such compromise can actually lead to this relationship becoming fractured.

The findings of this study, as with others that precede it [6,39,40], underscore the need for unbiased, high quality information about medical tourism to be made accessible to all those who have a potential stake in this global health services practice. Consideration must be given to who is in a position to create trustworthy information, how that information should be disseminated, and how it can be kept up to date. The findings highlight concern and confusion around family physicians’ responsibilities to medical tourists in their practices, and highlight the fact that informational interventions are needed to clarify home country liability on advising prospective medical tourists about destination clinics and for caring for returning medical tourists. Finally, the findings also suggest that greater physician introspection about personal biases regarding medical tourism will yield benefits to the trusting relationship they share with patients.

Canada has a national public health care system and family physicians in this system serve as gatekeepers to secondary and tertiary care [19,41]. Future studies of markedly different health system contexts, such as highly privatized systems, are key to the unfolding trust story we introduce in this analysis. Moreover, not all medical tourism procedures or patients are alike, regardless of similarities in infrastructure for health care. For example, in the case of patients facing debilitating or life-limiting illnesses who seek unproven interventions abroad, such as stem cells and chronic cerebrospinal venous insufficiency procedures, physicians must ground patients’ hopes for therapeutic solutions in current clinical realities [42]. In instances of patients obtaining transplants that involve purchased organs abroad, physicians must balance their moral beliefs against their legal and practical responsibilities [43,44]. Procedure-specific concerns may raise new issues of trust or distrust that impact the ongoing physician-patient relationship that should be explored in future research.

## Conclusions

Conversations about medical tourism raise salient concerns about trust and distrust that impact the physician-patient relationship that medical tourists have with their regular physicians at home. Unique tensions surface as Canadian family physicians must provide counsel about medical tourism as experts, in the face of significant deficits of reliable information. They must facilitate patient decision-making while hoping to distance themselves from patients’ decisions to engage in medical tourism. They must also manage fiduciary responsibilities both as gatekeepers of health care for patients and as agents of health care systems. Such tensions necessitate the provision of centralized and reliable information, and the development of clear policy surrounding liability. Pragmatic responsiveness will empower physicians as they walk the fine line of trust and distrust in the ever-changing landscape of biomedicine today.

## Endnotes

^a^In this article we use the word ‘patient’ as it was the term most commonly used by study participants when referring to those they treated in their capacities as family physicians.
